# Body Fat and Breast Cancer Risk in Postmenopausal Women: A Longitudinal Study

**DOI:** 10.1155/2013/754815

**Published:** 2013-04-07

**Authors:** Thomas E. Rohan, Moonseong Heo, Lydia Choi, Mridul Datta, Jo L. Freudenheim, Victor Kamensky, Heather M. Ochs-Balcom, Lihong Qi, Cynthia A. Thomson, Mara Z. Vitolins, Sylvia Wassertheil-Smoller, Geoffrey C. Kabat

**Affiliations:** ^1^Department of Epidemiology and Population Health, Albert Einstein College of Medicine, Bronx, NY 10461, USA; ^2^Karmanos Cancer Center, Department of Surgery, Wayne State University School of Medicine, Detroit, MI 48201, USA; ^3^Department of Social Sciences and Health Policy, Wake Forest School of Medicine, Winston-Salem, NC 27157, USA; ^4^Department of Social and Preventive Medicine, University at Buffalo, Buffalo, NY 14214, USA; ^5^Department of Public Health Sciences, University of California Davis, Davis, CA 95616, USA; ^6^Mel and Enid Zuckerman College of Public Health, University of Arizona, Tucson, AZ 85724, USA; ^7^Department of Epidemiology and Prevention, Wake Forest School of Medicine, Winston-Salem, NC 27127, USA

## Abstract

Associations between anthropometric indices of obesity and breast cancer risk may fail to capture the true relationship between excess body fat and risk. We used dual-energy-X-ray-absorptiometry- (DXA-) derived measures of body fat obtained in the Women's Health Initiative to examine the association between body fat and breast cancer risk; we compared these risk estimates with those for conventional anthropometric measurements. The study included 10,960 postmenopausal women aged 50–79 years at recruitment, with baseline DXA measurements and no history of breast cancer. During followup (median: 12.9 years), 503 incident breast cancer cases were diagnosed. Hazard ratios (HR) and 95% confidence intervals (CI) were estimated using Cox proportional hazards models. All baseline DXA-derived body fat measures showed strong positive associations with breast cancer risk. The multivariable-adjusted HR for the uppermost quintile level (versus lowest) ranged from 1.53 (95% CI 1.14–2.07) for fat mass of the right leg to 2.05 (1.50–2.79) for fat mass of the trunk. Anthropometric indices (categorized by quintiles) of obesity (BMI (1.97, 1.45–2.68), waist circumference (1.97, 1.46–2.65), and waist : hip ratio (1.91, 1.41–2.58)) were all strongly, positively associated with risk and did not differ from DXA-derived measures in prediction of risk.

## 1. Introduction

Obesity is defined as an excess accumulation of adipose tissue that results when calorie intake exceeds energy expenditure [1]. Obesity can be assessed in a number of ways, including anthropometrically, using body mass index (BMI) (weight (kg)/height (m^2^)), waist circumference, or waist : hip ratio (waist circumference (cm) divided by hip circumference (cm)), or by using techniques that directly measure body fat, such as dual energy X-ray absorptiometry (DXA), bioelectrical impedance analysis, computed tomography, and magnetic resonance imaging [2, 3]. Anthropometric approaches are usually used in epidemiologic studies of the association between obesity and disease risk because they are relatively inexpensive and easy to implement.

Using anthropometric approaches, and in particular BMI, many epidemiologic studies have shown that obesity is associated with increased risk of breast cancer in postmenopausal women [4–6]. However, a major limitation of BMI as an index of obesity is that the numerator (i.e., weight) fails to differentiate between lean and fat mass [3], so that two individuals with the same BMI may differ considerably with respect to percent body fat [7]. Differences between individuals in terms of age, sex, and/or ethnicity further complicate the interpretation of BMI [8]. Other anthropometric indices such as waist circumference and waist : hip ratio are indirect measures of central adiposity [8] rather than of whole body fat distribution [9]. For these reasons, associations between BMI and other anthropometric indices and breast cancer risk may fail to capture the true nature of the relationship between excess body fat and risk.

Given the limitations of anthropometric indices, studies that employ direct measures of body fat may yield more accurate estimates of the association between adiposity/obesity and breast cancer risk. DXA directly assesses bone mineral content and the soft tissue surrounding the bone by measuring the amounts of fat and lean tissue, and it can be used to both characterize body composition and provide precise estimates of fat, bone, and bone-free lean mass [10]. Therefore, we used DXA-derived measures of body fat obtained in the Women's Health Initiative (WHI) [11] to examine the association between body fat and risk of incident, invasive breast cancer. Additionally, we compared these risk estimates with those obtained using conventional anthropometric measurements.

## 2. Materials and Methods

### 2.1. Setting

The WHI is a large, multicenter study designed to foster understanding of the determinants of major chronic diseases in postmenopausal women. Women between the ages of 50 and 79 and representing major racial/ethnic groups were recruited from the general population at 40 clinical centers throughout the United States between 1993 and 1998 into either the Clinical Trial (CT) component (*n* = 68,132) or the Observational Study (OS) component (*n* = 93,676) [12]. The CT component included several randomized controlled interventions: the hormone therapy (HT) trials of estrogen alone and of estrogen plus progestin, the low-fat dietary modification (DM) trial, and the calcium-vitamin D supplementation (CAD) trial, none of which was designed to promote change in body weight. Details of the design and reliability of the baseline measures have been published elsewhere [12, 13]. Written informed consent was obtained from all participants and the WHI was approved by the institutional review boards of all participating institutions. The analyses reported here were based on the cohort of 11,393 women who had body fat assessed using DXA.

### 2.2. Data Collection and Variable Definition

Self-administered questionnaires, completed at study entry, were used to collect information on demographics, medical, reproductive, and family history, and on dietary and lifestyle factors, including smoking history, alcohol consumption, and recreational physical activity [14]. In addition, all participants had their weight, height, and waist and hip circumferences measured at baseline by trained staff using standardized protocols; during followup, the majority had measurements taken at years three and six, and small proportions had measurements taken at years one and nine. Weight was measured to the nearest 0.1 kg, and height to the nearest 0.1 cm. Waist circumference at the natural waist or narrowest part of the torso and hip circumference at the maximal circumference were recorded to the nearest 0.1 cm. BMI was computed as weight (kilograms (kg)) divided by the square of height (meters (m)).

### 2.3. Body Fat Measurements

In the WHI, 11,393 women in three designated centers (Birmingham, Tucson/Phoenix, and Pittsburgh) had body fat measured by whole body DXA scans performed in fan-beam mode and obtained from Hologic QDR scanners (QDR 2000, 2000+, or 4500) (Hologic, Inc., Waltham, MA). Measurements were made at baseline and again at years one, three, six, and nine of followup. A standardized procedure for participant positioning and scan analysis was executed for all scans in the three centers. All DXA operators attended a central training session and were certified on the basis of an evaluation of scanning and analysis technique. Spine and whole body phantom scans were used to monitor scanner performance longitudinally [10]. Quality control entailed having technicians at the DXA coordinating center (University of California, San Francisco) review unacceptable scans, outliers, and a random sample of all scans. When two QDR2000 scanners were retired, *in vivo* cross-calibration was performed at two sites to convert QDR4500 to QDR2000-equivalent values [10]. These correction factors and adjustments for longitudinal changes in scanner performance were applied to participant scan results.

### 2.4. Ascertainment of Outcome

Clinical outcomes (including new cancer diagnoses) were updated semiannually in the CT and annually in the OS using in-person, mailed, or telephone questionnaires. Self-reports of breast cancer were verified by centralized review of medical records and pathology reports by trained physician adjudicators [15]. As of March 31, 2011, after a median of 12.9 years of followup, a total of 537 incident cases of breast cancer had been diagnosed among the 11,393 subjects with DXA measurements. Less than 1% of the cohort was lost to followup. 

### 2.5. Available Data

A total of 11,393 women had DXA measurements performed during the course of the study. Of these, we excluded 108 women (8 cases and 100 noncases) who were missing baseline DXA measurements and 325 women (26 cases and 299 noncases) with a previous history of breast cancer. This left 10,960 women available for analysis (503 cases and 10,457 noncases), of whom 380 (76.5%) cases and 7628 (72.9%) noncases were not in any intervention group (OS, control, placebo), 6 (1.2%) cases and 289 (2.8%) noncases were in the estrogen-alone trial intervention group and not in any other, 19 (3.8%) cases and 314 (3.0%) noncases were in the estrogen plus progestin trial intervention group and not in any other, 50 (9.9%) cases and 943 (9.0%) noncases were in the dietary modification trial intervention group and not in any other, 26 (5.2%) cases and 644 (6.2%) noncases were in the calcium plus vitamin D supplementation trial intervention group and not in any other, 22 (4.4%) cases and 612 (5.9%) noncases were in the intervention groups of 2 trials, and 0 cases and 27 (0.3%) noncases were in the intervention groups of 3 trials. 

### 2.6. Statistical Analysis

Cox proportional hazards models were used to estimate hazard ratios (HRs) and 95% confidence intervals (CI) for the associations between various measures of body fat (see below) and risk of breast cancer. The outcome was time to breast cancer in days from enrollment. Cases contributed person-time to the study from their date of enrollment until the date of diagnosis of their breast cancer, and noncases contributed person-time from their date of enrollment and were censored as of the end of followup (March 31, 2011), the date of death, or the date of withdrawal from the study, whichever came first. 

In the first stage of the analysis, baseline body fat measures (whole body fat mass (g), whole body percent fat, fat mass of trunk (g), fat mass of right leg (g), fat mass of left leg (g), and ratio of fat mass of trunk to average of fat mass of right and left legs (as an estimate of upper abdominal fat versus hip fat)) and anthropometric indices of obesity (BMI (kg/m^2^), waist circumference (cm), and waist : hip ratio) were used as the exposure variables, and they were categorized by quintiles. In multivariate analyses, adjustment was made for established risk factors for breast cancer and for randomization assignment in the CT. Specifically, we adjusted for age (years) at baseline (continuous), family history of breast cancer (yes, no), age (years) at menarche (<12, 12, 13, >13), age (years) at first full-term pregnancy (<20, 20–29, ≥30, missing), parity (0, 1, 2, 3, 4, ≥5), age (years) at menopause (<45 years, 45–54, ≥55, missing), metabolic equivalents (METs) from physical activity (MET hours/week, continuous—calculated as described elsewhere [14]), history of breast biopsy (yes, no), oral contraceptive use (ever, never), hormone therapy (type and duration), alcohol intake (servings per week—continuous), pack-years of smoking (continuous), education (less than high school graduate, high school graduate/some college, college graduate, postcollege), ethnicity (White, Black, other), randomization assignment in the CT, and total caloric intake (kcal/day—continuous). Trial assignment was taken into account by including dummy variables for each arm of each trial (i.e., intervention, placebo, control) in the multivariable model. Tests for interaction (with HT use) were based on likelihood ratio tests comparing models with and without product terms representing the variables of interest, and tests for trend were performed by assigning the median value to each category of the exposure variable of interest and modeling this variable as a continuous variable. The proportional hazards assumption—evaluated by testing the correlation of Schoenfeld residuals of predictors with ranked survival times and by testing the interaction between the predictors of interest and log-survival times—was not violated. All *P* values were two sided; *P* values < 0.05 were considered statistically significant.

In the second stage of the analysis, we analyzed the repeated body fat measurements and anthropometric indices as time-dependent covariates in Cox proportional hazards models to account for fluctuations in the measurements over time [16]. With this approach, the predictive significance of various aspects of these measures was evaluated, including time-lagged values (1–3 years, 2–4 years, and 3–5 years prior to diagnosis of breast cancer) and the average of all measurements. The relevant time-dependent covariate for subjects at risk at time *t* was a function of measurements obtained only until the time of diagnosis in the index case. Among cases, measurements made within 1 year of diagnosis (*n* = 35 for DXA-derived body fat measurements; *n* = 28 for anthropometric variables) were excluded from all analyses since these values may have been influenced by the presence of subclinical disease. 

Finally, in order to assess the predictive power of whole body fat mass and percent body fat versus BMI, of fat mass of the trunk versus waist circumference, and of the trunk : leg fat mass ratio versus waist : hip ratio, we computed *c*-indices (the *c*-index is a measure of the discriminatory ability of a given survival model) [17] by applying a SAS macro developed by the Mayo Clinic Biostatistics Group [18]. To test for differences between the *c*-indices from the different models we applied an *ad hoc* asymptotic *z*-test statistic assuming that the correlations between the *c*-indices are 0.5. The *z* scores were computed as (*c*1-*c*2)/sqrt(se^2^(*c*1) + se^2^(*c*2) − se(*c*1)*se(*c*2)), where *c*1 and *c*2 are estimated *c*-indices and se(*c*1) and se(*c*2) are their corresponding standard errors.

## 3. Results

The baseline DXA-derived body fat measurements and the anthropometric measurements were mostly moderately to strongly positively correlated with each other (Table 1). However, the correlations with waist : hip ratio were generally weaker than those with the other variables, and there were inverse associations between fat mass of the right and left legs with the ratio of trunk fat mass to the average of the fat mass of the right and left legs.

DXA-derived whole body fat mass was positively associated with a history of diabetes, a relatively early menarche, intake of energy, and carbohydrate, and with Black race and (to a lesser extent) Hispanic ethnicity, and was inversely associated with ever use of hormone therapy (HT), and with alcohol intake and physical activity (Table 2). For BMI, the patterns of associations with these variables were similar to those for DXA-derived whole body fat mass (data not shown).

All baseline DXA-derived body fat measures showed strong positive associations with breast cancer risk (Table 3). The multivariable-adjusted HR for the uppermost quintile level ranged from 1.53 (95% CI 1.14–2.07) for fat mass of the right leg to 2.05 (95% CI 1.50–2.79) for fat mass of the trunk. These results changed little when we additionally adjusted for scanner type, a personal history of diabetes, height, carbohydrate intake, and fat intake (data not shown). 

Data on estrogen receptor (ER) status were available for 437 cases (353 ER+, 84 ER−). The DXA-derived body fat variables were all strongly positively associated with risk of ER+ breast cancer, but were not associated with risk of ER− breast cancer, and these estimates differed significantly from each other (data not shown). There were too few cases to allow meaningful analyses by combined ER/progesterone receptor status.

When we stratified by ever use of HT, strong positive associations, of similar magnitude to those presented in Table 3, were evident in both ever and never users (data not shown). The HRs for whole body fat mass, whole body percent fat, and fat mass of the right and left legs were slightly higher in never users than in ever users, whereas the reverse was observed for trunk fat. In contrast, the HRs for the ratio of trunk fat mass : leg fat mass were substantially higher in never users than in ever users: HR (95%CI)_quintile  5 versus quintile  1_ = 2.55 (1.53–4.25) in never users and 1.37 (0.90–2.10) in ever users. However, on formal testing, there was no evidence of effect modification by HT use.

In sensitivity analyses, the patterns shown in Table 3 were similar after exclusion of those in the intervention group of the dietary modification trial (these subjects experienced slight weight loss during the followup period [19]); after exclusion of those in the intervention groups of the HT trials (HT may attenuate changes in body composition with age (e.g., gain in percent fat mass and upper body fat [20])); after excluding the nine cases who were diagnosed with ductal carcinoma *in situ* of the breast prior to the development of invasive breast cancer; and after excluding the first three years of followup (to address the possibility of reverse causality) (data not shown). However, the results were somewhat attenuated after exclusion of the 83 cases and 1264 noncases who were very obese (BMI > 35 kg/m^2^) (accurate measurement of body composition is difficult in obese women [21, 22]). Specifically, although the HR estimates for the second to fifth quintile levels were all greater than one, they were generally lower than the comparable estimates for the entire study population, and the trends for whole body percent fat (*P* = 0.12), and for fat mass of the right (*P* = 0.12) and left (*P* = 0.08) legs, were no longer statistically significant. The HRs (95% CIs) for the fifth versus the first quintile level were 1.47 (0.99–2.17) for whole body fat mass, 1.29 (0.89–1.87) for whole body percent fat, 1.69 (1.15–2.47) for trunk fat mass, 1.24 (0.86–1.78) for fat mass of the right leg, 1.40 (0.98–1.99) for fat mass of the left leg, and 1.75 (1.25–2.44) for the ratio of trunk fat mass : average of right and left leg fat mass.

All three anthropometric measures (BMI, waist circumference, waist : hip ratio) were strongly, positively associated with breast cancer risk (Table 4). The multivariable-adjusted HRs (95% CIs) for the highest versus the lowest quintile levels were 1.97 (1.45–2.68) for BMI, 1.97 (1.46–2.65) for waist circumference, and 1.91 (1.41–2.58) for waist : hip ratio, and the associated trends in risk were statistically significant. These associations were evident in both ever and never users of HT and there was no evidence of effect modification by HT use (data not shown). When both waist : hip ratio and BMI were included in the same model (together with other variables included in the multivariate models), there was some attenuation of the associations, but both remained significant: the HRs (95% CI) for the 2nd to 5th quintile levels for waist : hip ratio were 1.31 (0.97–1.78), 1.34 (0.98–1.83), 1.12 (0.80–1.56), and 1.59 (1.15–2.19), respectively, with *p *(trend) = 0.01, and those for BMI were 1.09 (0.79–1.49), 1.30 (0.95–1.78), 1.26 (0.91–1.75), and 1.73 (1.24–2.40), respectively, with *p *(trend) = 0.0003.

Of the 10,960 women who had baseline DXA measurements, 4,360 (40%) had measurements at year one (OS participants were not required to have DXA measurements at this time point), 8,906 (81%) at year three, 7,822 (71%) at year six, and 3,972 (36%) at year nine.  Table 5 shows the results of the time-dependent covariate analyses. For the average of all measurements, the results were similar to those obtained using the baseline measurements, with those for trunk fat showing the strongest association, followed by those for whole body fat mass. The association of body fat measured in different time windows was also strongest for fat mass of the trunk and for whole body fat mass, both of which showed statistically significant positive associations and trends in all time windows. The magnitude of the associations was similar for measurements made 1–3 years and 2–4 years prior to diagnosis and was somewhat stronger for those made 3–5 years prior to diagnosis. 

Time-dependent covariate analyses of the anthropometric measures of obesity yielded strong, positive associations with breast cancer risk (Table 6). However, the point estimates were slightly attenuated compared with those obtained using the baseline measurements only. As with the time-dependent analyses of the DXA-derived measures, the magnitude of the associations was strongest for the measurements made 3–5 years prior to diagnosis (data not shown). 

The *c*-indices for the DXA and anthropometric variables of interest were all very similar, ranging from 0.630 for BMI to 0.645 for waist circumference (Table 7). Furthermore, none of the pairwise comparisons, including whole body fat mass and percent body fat versus BMI, fat mass of the trunk versus waist circumference, and the trunk : leg fat mass ratio versus waist : hip ratio, was statistically significant. Additionally, the *c*-index for a model with both WHR and BMI was 0.647, which did not significantly improve on the *c*-index for a model with BMI alone (0.641) or that for a model with WHR alone (*c* = 0.640). Similarly, fitting a model with WHR and total leg fat mass yielded a *c*-statistic of 0.649, which did not represent a significant improvement over those for WHR alone or total leg fat mass alone (0.636). Similar results were observed when we modeled WHR and fat mass of the right leg and WHR and fat mass of the left leg (data not shown).

## 4. Discussion

The results of the present study provide strong support for a positive association between body fat and breast cancer risk in postmenopausal women. The DXA-derived measures of body fat and fat distribution were associated with 1.5- to 2-fold increases in breast cancer risk (for the highest versus the lowest quintile level). The point estimates were strongest for fat mass of the trunk and for whole body fat mass, but the confidence intervals for these associations overlapped with those for all other body fat measures. There was no evidence of effect modification by ever use of HT, and the results were generally robust to various sensitivity analyses. However, the results were attenuated somewhat after exclusion of the very obese (BMI > 35 kg/m^2^). The results of the repeated measures analyses generally supported the findings from the analysis of the baseline measures. Finally, anthropometric indices of obesity were also strongly associated with increased breast cancer risk and they predicted risk as well as the DXA-derived measures of body fat.

DXA provides highly reproducible and accurate measures of body fat, and therefore it is now used extensively for estimating body composition [8]. It allows regional analysis of fat distribution, and although it does not differentiate visceral fat from subcutaneous fat in the abdominal region [23], there is a strong correlation between trunk fat measured by DXA and abdominal visceral fat measured by single slice computerized tomography [24]. Variability in DXA measurements can arise because of differences between scanner models and between different versions of software from the same manufacturer [8, 23]. In this regard (as indicated earlier), in the present study, when two QDR2000 scanners were retired, *in vivo* cross-calibration was performed to convert QDR4500 to QDR2000-equivalent values, and these correction factors and adjustments for longitudinal changes in scanner performance were applied to participant scan results.

We are not aware of any previous studies of DXA-derived measures of body fat and risk of breast cancer. Nevertheless, several previous case-control [25] and cohort studies [26–29] have shown direct measures of body fat, obtained using methods other than DXA, to be positively associated with breast cancer risk. In two of these studies the association was observed with upper body fat [25] or central adiposity [26], while in the remaining studies, the associations were observed with body fat overall [27–29]. However, these studies were based on fewer cases (106 [26] to 357 [29]) than the present report, did not have repeated measures of body fat, and used either skinfold thickness [25, 26] or bioelectrical impedance analysis (BIA) [27–29] to measure body fat. Both of these approaches to measuring body fat have limitations: skinfold thicknesses are measured with greater error than other anthropometric measures and are unreliable measures of central adiposity, while BIA, in comparison with DXA, overestimates percent body fat in lean subjects and underestimates it in obese subjects [30].

The associations between the DXA measures of body fat and breast cancer risk appeared to be confined largely to ER+ tumors. However, given the relatively small number of ER− cases, this finding warrants cautious interpretation. Nevertheless, it is in accord with previous observations [31, 32], and it has been suggested that this may result from an obesity-induced increase in circulating free estrogen levels which induce progesterone receptor expression and hence stimulation of ER+/PR+ tumors [32]. 

The present study also showed that anthropometric indices of obesity, namely, BMI, waist circumference, and waist : hip ratio, were all strongly, positively associated with breast cancer risk. These findings are essentially in accord with those of previous studies conducted elsewhere [4–6]. Previous investigations in WHI have also shown positive associations with BMI [33–35], although in two of these [33, 35] the association was confined largely to those who had never used HT, and in one [35] there was no association with waist : hip ratio. The present WHI study showed further that these indices did not differ in their ability to predict risk when compared with DXA-derived measures of whole body fat mass/percent body fat, fat mass of the trunk, and trunk : leg fat mass ratio, respectively. In this regard, as in the present study, BMI has been shown in previous studies to have a relatively strong positive correlation with measures of percent body fat obtained using DXA [7, 36, 37] or BIA [38, 39], while waist circumference and waist : hip ratio, which are used as indirect measures of abdominal/central obesity, have been shown to have relatively strong positive correlations with total abdominal fat and abdominal visceral fat measured using computerized tomography [40] or magnetic resonance imaging [41]; waist circumference, but not waist : hip ratio, has also been shown to have strong positive correlations with percent body fat and total fat mass measured using DXA [42]. However, BMI, the most widely used anthropometric index of obesity, has been shown to have low sensitivity for diagnosing obesity defined according to percent body fat measured using body composition measurement techniques such as DXA, BIA, and air displacement plethysmography, such that substantial proportions of individuals classified as lean or overweight by BMI are classified as obese according to their percent body fat [3, 43]. Consistent with this observation, such individuals have a higher prevalence of dysregulation of metabolic factors (e.g., glucose, insulin, insulin resistance, C-reactive protein, etc.) and of the associated metabolic syndrome compared to lean individuals with normal body fat amounts [43, 44]. Recent evidence from WHI provides some support for an association between the metabolic syndrome and its component parts and breast cancer risk [45]. These observations suggest that while use of BMI may allow reasonably accurate prediction of breast cancer risk for groups of individuals, it provides potentially misleading information about the risk for a given individual, with implications for clinical management, particularly of those with normal weight obesity (i.e., normal body weight based on BMI but relatively high body fat content) [44].

There is now a substantial body of epidemiologic evidence supporting a positive association between obesity in postmenopausal women and breast cancer risk. In general terms, it has been suggested that the association of obesity with increased cancer risk may result from alterations in adipocyte biology or through alterations in the stromal-vascular fraction of adipose tissue [1]. Hence, many possible mechanisms for the increase in risk have been proposed, including increased circulating levels of leptin and inflammatory cytokines (e.g., TNF*α*, IL-6, PAI1) and decreased adiponectin levels, as well as increased insulin/IGF signaling, elevated lipid levels, and, particularly in relation to breast cancer in postmenopausal women, an increase in aromatase expression in adipose tissue and a decrease in circulating sex hormone-binding globulin resulting (as mentioned earlier) in elevated bioavailable estradiol levels [1, 46, 47]. However, these mechanisms may not fully explain the relationship between obesity and cancer risk, and other mechanisms, including obesity-induced hypoxia, shared genetic susceptibility between obesity and cancer, and migration of adipose stromal cells might also be relevant [48]. 

The present study has several strengths including its prospective design, long-term followup, large number of breast cancer cases, essentially complete ascertainment of cases and centralized pathology review, standardized protocols administered by trained staff for measurement of anthropometric variables and of body fat using DXA, repeated measures of the variables of interest over time, and the availability of information on a wide range of potential confounding factors. Limitations of the study include the fact that it was restricted to postmenopausal women, so that there were no DXA measures of body fat from earlier in life, and the limitations of DXA itself, including between-model variability in DXA measurements, although the latter issue was addressed by *in vivo* cross-calibration.

## 5. Conclusion

In conclusion, the results of the present study provide strong support for a positive association between DXA-derived measures of body fat (whole body fat mass, percent body fat, fat mass of the trunk and legs, and the ratio of trunk to leg fat mass) and breast cancer risk in postmenopausal women. They suggest further that anthropometric indices of obesity (BMI, waist circumference, and waist : hip ratio) predict breast cancer risk as well as DXA-derived measures of body fat.

## Figures and Tables

**Table 1:** Correlation matrix of baseline DXA and anthropometric measures among non-cases (*N* = 10,457)^a^.

	% body fat	Trunk fat mass	Fat mass left leg	Fat mass right leg	Ratio of trunk to leg fat mass	BMI	Waist circum-ference	Waist : hip ratio
Whole body fat mass	0.88	0.95	0.88	0.89	0.28	0.91	0.84	0.29
% body fat		0.86	0.77	0.77	0.31	0.75	0.68	0.24
Trunk fat mass			0.71	0.72	0.53	0.87	0.87	0.42
Fat mass left leg				0.99	−0.15	0.77	0.60	0.01
Fat mass right leg					−0.15	0.77	0.60	0.02
Ratio of trunk to leg fat mass						0.31	0.50	0.58
BMI							0.84	0.35
Waist circumference								0.68

^a^ *P*-values for correlations were <0.0001, except for fat mass of the right leg with waist : hip ratio (*P* = 0.20) and fat mass of the left leg with waist : hip ratio (*P* = 0.07).

**Table 2:** Distribution at baseline of selected characteristics by quintiles of DXA-derived whole body fat mass among non-cases (*N* = 10,457).

	Quintiles of whole body fat mass				
	1	2	3	4	5
*N*	2113	2098	2095	2086	2065
Age, mean	63.5	63.7	64.0	63.3	61.8
Family history of breast cancer (%)	15.9	15.7	16.6	15.2	15.6
History of diabetes (%)	2.8	3.6	6.5	10.0	16.4
Age at menarche (% <12 yrs)	15.3	19.3	20.1	23.2	26.3
Age at first full-term pregnancy (% ≥30 yrs)	8.7	7.6	6.7	6.6	7.7
Parity (mean)	2.4	2.5	2.7	2.8	2.8
Age at menopause, mean	47.3	46.3	46.4	46.1	45.4
Oral contraceptive use (% ever)	38.1	37.3	35.9	36.7	38.3
Hormone therapy (% ever)	58.7	57.7	53.0	50.5	44.5
Alcohol intake—drink/wk (mean)	2.3	2.0	1.8	1.4	1.0
Physical activity (MET), mean	15.7	11.2	10.2	7.7	6.2
Pack-years of smoking, mean	8.7	9.4	8.9	8.4	9.5
Total energy intake (Kcal/day), mean	1528	1585	1632	1709	1848
Total carbohydrate intake (g/day), mean	198	199	201	207	217
Education (% post-college)	29.1	22.5	19.5	19.3	17.6
Ethnicity, %					
Non-Hispanic white	86.6	81.4	77.6	73.9	67.9
Black	7.6	10.6	13.5	15.8	22.6
Hispanic	3.7	6.6	7.2	7.9	6.7
Other	2.1	1.4	1.7	2.4	2.8

**Table 3:** Hazard ratios (HR) and 95% confidence intervals (CI) for the association of DXA-derived body fat measured at baseline with risk of incident, invasive breast cancer in postmenopausal women.

	Quintiles					*P* for trend
	1	2	3	4	5	
Whole body fat mass						
Cases	77	87	112	106	121	
Person-years	25,490	25,188	25,404	24,935	24,511	
Age- and energy-adjusted HR (95% CI)	1.00 Ref.	1.14 (0.84–1.56)	1.47 (1.10–1.96)	1.42 (1.06–1.91)	1.69 (1.27–2.25)	0.0001
Multivariable-adjusted HR (95% CI)^a^	1.00 Ref.	1.18 (0.86–1.62)	1.57 (1.16–2.13)	1.47 (1.08–2.02)	1.88 (1.38–2.57)	<0.0001

Whole body percent fat						
Cases	81	96	112	100	114	
Person-years	25,730	25,157	25,137	25,141	24,364	
Age- and energy-adjusted HR (95% CI)	1.00 Ref.	1.21 (0.90–1.62)	1.41 (1.06–1.88)	1.26 (0.94–1.69)	1.50 (1.13–1.99)	0.007
Multivariable-adjusted HR (95% CI)^a^	1.00 Ref.	1.21 (0.89–1.64)	1.48 (1.10–1.99)	1.28 (0.94–1.75)	1.60 (1.18–2.18)	0.004

Fat mass of trunk						
Cases/person-years	79	94	97	106	127	
Person-years	25,770	25,353	25,252	24,689	24,466	
Age- and energy-adjusted HR (95% CI)	1.00 Ref.	1.21 (0.89–1.63)	1.24 (0.92–1.67)	1.41 (1.05–1.88)	1.74 (1.31–2.31)	<0.0001
Multivariable-adjusted HR (95% CI)^a^	1.00 Ref.	1.31 (0.96–1.78)	1.34 (0.98–1.83)	1.56 (1.15–2.14)	2.05 (1.50–2.79)	<0.0001

Fat mass of right leg						
Cases	81	86	107	109	120	
Person-years	24,666	25,231	25,522	25,276	24,835	
Age- and energy-adjusted HR (95% CI)	1.00 Ref.	1.05 (0.78–1.43)	1.30 (0.98–1.74)	1.34 (1.01–1.79)	1.53 (1.15–2.04)	0.0008
Multivariable-adjusted HR (95% CI)^a^	1.00 Ref.	1.00 (0.73–1.36)	1.26 (0.93–1.69)	1.29 (0.95–1.74)	1.53 (1.14–2.07)	0.001

Fat mass of left leg						
Cases	78	91	109	101	124	
Person-years	24,586	25,501	25,321	25,386	24,736	
Age- and energy-adjusted HR (95% CI)	1.00 Ref.	1.14 (0.84–1.54)	1.38 (1.03–1.85)	1.28 (0.95–1.72)	1.64 (1.24–2.19)	0.0004
Multivariable-adjusted HR (95% CI)^a^	1.00 Ref.	1.05 (0.77–1.43)	1.33 (0.99–1.79)	1.19 (0.87–1.62)	1.63 (1.21–2.20)	0.0007

Ratio of trunk fat mass to average of R and L leg fat mass						
Cases	74	117	104	104	104	
Person-years	25,932	25,391	25,388	24,864	23,955	
Age- and energy-adjusted HR (95% CI)	1.00 Ref.	1.61 (1.20–2.15)	1.43 (1.06–1.93)	1.45 (1.08–1.96)	1.51 (1.12–2.04)	0.04
Multivariable-adjusted HR (95% CI)^a^	1.00 Ref.	1.68 (1.25–2.28)	1.51 (1.10–2.06)	1.60 (1.17–2.18)	1.77 (1.29–2.42)	0.004

^a^Adjusted for age at enrollment, education, ethnicity, family history of breast cancer, age at menarche, age at first full-term birth, parity, age at menopause, oral contraceptive use, duration of hormone therapy, previous breast biopsy, physical activity, alcohol intake, pack-years of smoking, intake of energy, and randomization status.

**Table 4:** Hazard ratios (HR) and 95% confidence intervals (CI) for the association of body mass index, waist circumference, and waist-hip ratio with risk of incident, invasive breast cancer in postmenopausal women.

	Quintiles					*P* for trend
	1	2	3	4	5	
Body mass index						
Cases	80	89	102	102	129	
Person-years	25,409	25,468	25,058	24,868	24,109	
Age- and energy-adjusted HR (95% CI)	1.00 Ref.	1.13 (0.83–1.53)	1.31 (0.98–1.76)	1.33 (0.99–1.79)	1.74 (1.31–2.31)	<0.0001
Multivariable-adjusted HR (95% CI)^a^	1.00 Ref.	1.15 (0.84–1.57)	1.41 (1.04–1.91)	1.43 (1.05–1.95)	1.97 (1.45–2.68)	<0.0001

Waist circumference						
Cases	89	74	100	108	131	
Person-years	27,187	23,190	25,014	25,401	24,332	
Age- and energy-adjusted HR (95% CI)	1.00 Ref.	0.98 (0.72–1.43)	1.22 (0.91–1.63)	1.30 (0.98–1.72)	1.68 (1.28–2.21)	<0.0001
Multivariable-adjusted HR (95% CI)^a^	1.00 Ref.	1.00 (0.72–1.37)	1.27 (0.94–1.72)	1.44 (1.07–1.94)	1.97 (1.46–2.65)	<0.0001

Waist : hip ratio						
Cases	85	103	102	88	124	
Person-years	26,315	25,472	25,139	24,480	23,655	
Age- and energy-adjusted HR (95% CI)	1.00 Ref.	1.26 (0.95–1.69)	1.26 (0.94–1.68)	1.12 (0.83–1.51)	1.61 (1.22–2.13)	0.004
Multivariable-adjusted HR (95% CI)^a^	1.00 Ref.	1.39 (1.02–1.87)	1.46 (1.08–1.98)	1.30 (0.94–1.78)	1.91 (1.41–2.58)	0.0001

^a^Adjusted for age at enrollment, education, ethnicity, family history of breast cancer, age at menarche, age at first full-term birth, parity, age at menopause, oral contraceptive use, duration of hormone therapy, previous breast biopsy, physical activity, alcohol intake, pack-years of smoking, intake of energy, and randomization status.

**Table 5:** Hazard ratios (HR) and 95% confidence intervals (CI) from time-dependent covariate analysis of the association of DXA-derived body fat with risk of incident, invasive breast cancer in postmenopausal women.

	Quintiles					*P* for trend
	1	2	3	4	5	
*Average value*:						
Whole body fat mass						
Age- and energy-adjusted HR (95% CI)	1.00 Ref.	1.05 (0.76–1.44)	1.31 (0.97–1.77)	1.25 (0.92–1.70)	1.53 (1.13–2.07)	0.003
Multivariable-adjusted HR (95% CI)^a^	1.00 Ref.	1.04 (0.75–1.44)	1.37 (1.00–1.86)	1.29 (0.93–1.78)	1.60 (1.16–2.21)	0.002
Whole body percent fat						
Age- and energy-adjusted HR (95% CI)	1.00 Ref.	1.00 (0.73–1.37)	1.33 (0.99–1.78)	1.06 (0.78–1.44)	1.47 (1.10–1.97)	0.01
Multivariable-adjusted HR (95% CI)^a^	1.00 Ref.	0.99 (0.72–1.38)	1.37 (1.01–1.85)	1.08 (0.78–1.49)	1.52 (1.11–2.08)	0.01
Fat mass of trunk						
Age- and energy-adjusted HR (95% CI)	1.00 Ref.	1.24 (0.90–1.70)	1.21 (0.89–1.66)	1.46 (1.07–1.99)	1.82 (1.34–2.47)	<0.0001
Multivariable-adjusted HR (95% CI)^a^	1.00 Ref.	1.33 (0.96–1.85)	1.29 (0.93–1.79)	1.64 (1.19–2.27)	2.07 (1.49–2.87)	<0.0001
Fat mass of right leg						
Age- and energy-adjusted HR (95% CI)	1.00 Ref.	1.05 (0.77–1.43)	1.23 (0.91–1.65)	1.21 (0.90–1.64)	1.69 (1.27–2.26)	0.0003
Multivariable-adjusted HR (95% CI)^a^	1.00 Ref.	0.96 (0.70–1.32)	1.19 (0.88–1.61)	1.12 (0.82–1.54)	1.60 (1.18–2.16)	0.002
Fat mass of left leg						
Age- and energy-adjusted HR (95% CI)	1.00 Ref.	1.01 (0.74–1.38)	1.29 (0.96–1.73)	1.09 (0.81–1.48)	1.66 (1.25–2.21)	0.0008
Multivariable-adjusted HR (95% CI)^a^	1.00 Ref.	0.92 (0.67–1.27)	1.25 (0.93–1.69)	1.00 (0.73–1.37)	1.55 (1.15–2.09)	0.005
Ratio of trunk fat mass to average of R and L leg fat mass						
Age- and energy-adjusted HR (95% CI)	1.00 Ref.	1.43 (1.06–1.93)	1.24 (0.91–1.68)	1.21 (0.89–1.65)	1.37 (1.01–1.87)	0.24
Multivariable-adjusted HR (95% CI)^a^	1.00 Ref.	1.50 (1.10–2.04)	1.30 (0.94–1.79)	1.32 (0.95–1.82)	1.63 (1.18–2.25)	0.03

*Value 2–4 years prior to diagnosis*:						
Whole body fat mass						
Age- and energy-adjusted HR (95% CI)	1.00 Ref.	1.18 (0.80–1.74)	0.86 (0.56–1.30)	1.03 (0.69–1.53)	1.55 (1.06–2.25)	0.07
Multivariable-adjusted HR (95% CI)^a^	1.00 Ref.	1.16 (0.78–1.74)	0.94 (0.61–1.44)	1.04 (0.68–1.59)	1.67 (1.12–2.49)	0.04
Whole body percent fat						
Age- and energy-adjusted HR (95% CI)	1.00 Ref.	1.05 (0.70–1.56)	1.18 (0.81–1.73)	0.85 (0.56–1.28)	1.30 (0.89–1.90)	0.41
Multivariable-adjusted HR (95% CI)^a^	1.00 Ref.	1.07 (0.71–1.61)	1.22 (0.82–1.81)	0.88 (0.57–1.36)	1.35 (0.90–2.02)	0.33
Fat mass of trunk						
Age- and energy-adjusted HR (95% CI)	1.00 Ref.	1.31 (0.88–1.93)	0.91 (0.60–1.40)	1.28 (0.86–1.90)	1.60 (1.08–2.36)	0.04
Multivariable-adjusted HR (95% CI)^a^	1.00 Ref.	1.39 (0.93–2.09)	1.02 (0.66–1.59)	1.40 (0.92–2.14)	1.83 (1.21–2.79)	0.01
Fat mass of right leg						
Age- and energy-adjusted HR (95% CI)	1.00 Ref.	1.19 (0.81–1.76)	1.11 (0.75–1.65)	0.91 (0.60–1.39)	1.56 (1.07–2.26)	0.12
Multivariable-adjusted HR (95% CI)^a^	1.00 Ref.	1.11 (0.75–1.66)	1.08 (0.72–1.61)	0.85 (0.55–1.32)	1.50 (1.02–2.22)	0.17
Fat mass of left leg						
Age- and energy-adjusted HR (95% CI)	1.00 Ref.	1.17 (0.79–1.74)	1.24 (0.84–1.82)	0.95 (0.63–1.44)	1.50 (1.03–2.19)	0.14
Multivariable-adjusted HR (95% CI)^a^	1.00 Ref.	1.09 (0.73–1.62)	1.21 (0.82–1.80)	0.89 (0.58–1.36)	1.43 (0.96–2.13)	0.23
Ratio of trunk fat mass to average of R and L leg fat mass						
Age- and energy-adjusted HR (95% CI)	1.00 Ref.	1.28 (0.87–1.90)	1.14 (0.76–1.70)	1.20 (0.81–1.78)	1.15 (0.77–1.72)	0.69
Multivariable-adjusted HR (95% CI)^a^	1.00 Ref.	1.33 (0.89–2.00)	1.21 (0.79–1.83)	1.29 (0.85–1.95)	1.37 (0.90–2.09)	0.22

^a^Adjusted for age at enrollment, education, ethnicity, family history of breast cancer, age at menarche, age at first full-term birth, parity, age at menopause, oral contraceptive use, duration of hormone therapy, previous breast biopsy, physical activity, alcohol intake, pack-years of smoking, intake of energy, and randomization status.

**Table 6:** Hazard ratios (HR) and 95% confidence intervals (CI) from time-dependent covariate analysis of the association of body mass index, waist circumference, and waist-hip ratio with risk of incident, invasive breast cancer in postmenopausal women.

	Quintiles					*P* for trend
	1	2	3	4	5	
*Average value*:						
Body mass index						
Age- and energy-adjusted HR (95% CI)	1.00 Ref.	1.05 (0.76–1.44)	1.20 (0.89–1.63)	1.18 (0.87–1.61)	1.60 (1.19–2.14)	0.0012
Multivariable-adjusted HR (95% CI)^ a^	1.00 Ref.	1.05 (0.75–1.45)	1.29 (0.94–1.76)	1.25 (0.90–1.71)	1.74 (1.27–2.40)	0.0004
Waist circumference						
Age- and energy-adjusted HR (95% CI)	1.00 Ref.	0.89 (0.64–1.22)	0.99 (0.73-1.34)	1.28 (0.96–1.71)	1.56 (1.17–2.07)	<0.0001
Multivariable-adjusted HR (95% CI)^a^	1.00 Ref.	0.90 (0.65–1.25)	1.02 (0.74–1.40)	1.43 (1.05–1.94)	1.80 (1.32–2.45)	<0.0001
Waist : hip ratio						
Age- and energy-adjusted HR (95% CI)	1.00 Ref.	1.24 (0.91–1.69)	1.12 (0.82–1.53)	1.23 (0.91–1.68)	1.48 (1.10–2.00)	0.02
Multivariable-adjusted HR (95% CI)^a^	1.00 Ref.	1.38 (0.99–1.91)	1.28 (0.92–1.78)	1.46 (1.06–2.03)	1.79 (1.30–2.47)	0.0007

*Value 2–4 years prior to diagnosis*:						
Body mass index						
Age- and energy-adjusted HR (95% CI)	1.00 Ref.	0.96 (0.63–1.46)	1.06 (0.71–1.59)	0.97 (0.64–1.47)	1.43 (0.97–2.10)	0.08
Multivariable-adjusted HR (95% CI)^a^	1.00 Ref.	0.97 (0.63–1.50)	1.16 (0.77–1.77)	1.07 (0.69–1.65)	1.64 (1.08–2.49)	0.02
Waist circumference						
Age- and energy-adjusted HR (95% CI)	1.00 Ref.	0.82 (0.51–1.30)	0.61 (0.37–0.99)	0.92 (0.60–1.42)	1.27 (0.84–1.90)	0.17
Multivariable-adjusted HR (95% CI)^a^	1.00 Ref.	0.88 (0.55–1.41)	0.64 (0.38–1.07)	1.08 (0.69–1.71)	1.53 (0.98–2.37)	0.03
Waist : hip ratio						
Age- and energy-adjusted HR (95% CI)	1.00 Ref.	1.15 (0.72–1.84)	0.94 (0.57–1.52)	0.99 (0.62–1.60)	1.38 (0.89–2.14)	0.24
Multivariable-adjusted HR (95% CI)^a^	1.00 Ref.	1.23 (0.76–2.00)	1.06 (0.64–1.75)	1.14 (0.70–1.88)	1.66 (1.04–2.63)	0.05

^a^Adjusted for age at enrollment, education, ethnicity, family history of breast cancer, age at menarche, age at first full-term birth, parity, age at menopause, oral contraceptive use, duration of hormone therapy, previous breast biopsy, physical activity, alcohol intake, pack-years of smoking, intake of energy, and randomization status.

**Table 7:** *c*-indices for DXA and anthropometric measures.

Predictor	*c* (95% CI)	Difference from													
		WBF^a^		% body fat		Trunk FM		Total leg FM		Trunk : leg		BMI		WC	
		*z*	*p*	*z*	*p*	*z*	*p*	*z*	*p*	*z*	*p*	*z*	*p*	*z*	*p*
WBF	0.637 (0.610–0.663)														
% body fat	0.630 (0.603–0.656)	0.506	0.613												
Trunk FM	0.640 (0.614–0.666)	−0.269	0.788	0.773	0.439										
Total leg FM	0.636 (0.609–0.662)	−0.964	0.335	−1.479	0.139	−0.689	0.491								
Trunk : leg FM	0.635 (0.608–0.661)	0.157	0.875	−0.349	0.727	0.425	0.671	−1.125	0.260						
BMI	0.641 (0.615–0.668)	−0.335	0.738	−0.837	0.403	−0.067	0.947	−0.617	0.537	−0.491	0.624				
WC	0.645 (0.619–0.670)	−0.594	0.553	−1.101	0.271	−0.323	0.746	−0.363	0.717	−0.752	0.452	−0.255	0.799		
WHR	0.640 (0.615–0.665)	−0.236	0.813	−0.752	0.452	0.038	0.970	−0.745	0.456	−0.397	0.692	0.106	0.915	0.369	0.712

^a^WBF-whole body fat mass; trunk FM-fat mass of trunk; total leg FM-sum of fat mass of right leg and left leg; trunk : leg FM-ratio of trunk fat mass to average of R and L leg fat mass; BMI-body mass index; WC-waist circumference; WHR-waist : hip ratio.
